# Supplementary material for: SRC and TKS5 mediated podosome formation in fibroblasts promotes extracellular matrix invasion and pulmonary fibrosis
Source: Nat Commun. 2023 Sep 21;14:5882. doi: 10.1038/s41467-023-41614-x (PMC10514346; doi:10.1038/s41467-023-41614-x)
Supplement: Supplementary file 1 — Supplementary Information [file 41467_2023_41614_MOESM1_ESM.pdf]

SRC and TKS5 mediated podosome formation in fibroblasts promotes extracellular matrix invasion and pulmonary fibrosis

Ilianna Barbayianni[1,#], Paraskevi Kanellopoulou[1,#], Dionysios Fanidis[1], Dimitris Nastos[1], Eleftheria-Dimitra Ntouskou[1], Apostolos Galaris[1], Vaggelis Harokopos[1], Pantelis Hatzis[1], Eliza Tsitoura[2], Robert Homer[3], Naftali Kaminski[4], Katerina M. Antoniou[2], Bruno Crestani[5], Argyrios Tzouvelekis[6], and Vassilis Aidinis[1*]

[1]Institute for Fundamental Biomedical Research, Biomedical Sciences Research Center Alexander Fleming, Athens, Greece. [2]Department of Respiratory Medicine, School of Medicine, University of Crete, Heraklion, Greece. [3]Department of Pathology, Yale School of Medicine, New Haven CT, USA. [4]Department of Internal Medicine, Yale School of Medicine, New Haven CT, USA. [5]Department of Pulmonology, Bichat-Claude Bernard Hospital, Paris, France. [6]Department of Respiratory Medicine, School of Medicine, University of Patras, Patras, Greece.

[#]Equal contribution; [*]Correspondence: V.Aidinis@Fleming.gr

Online supplement

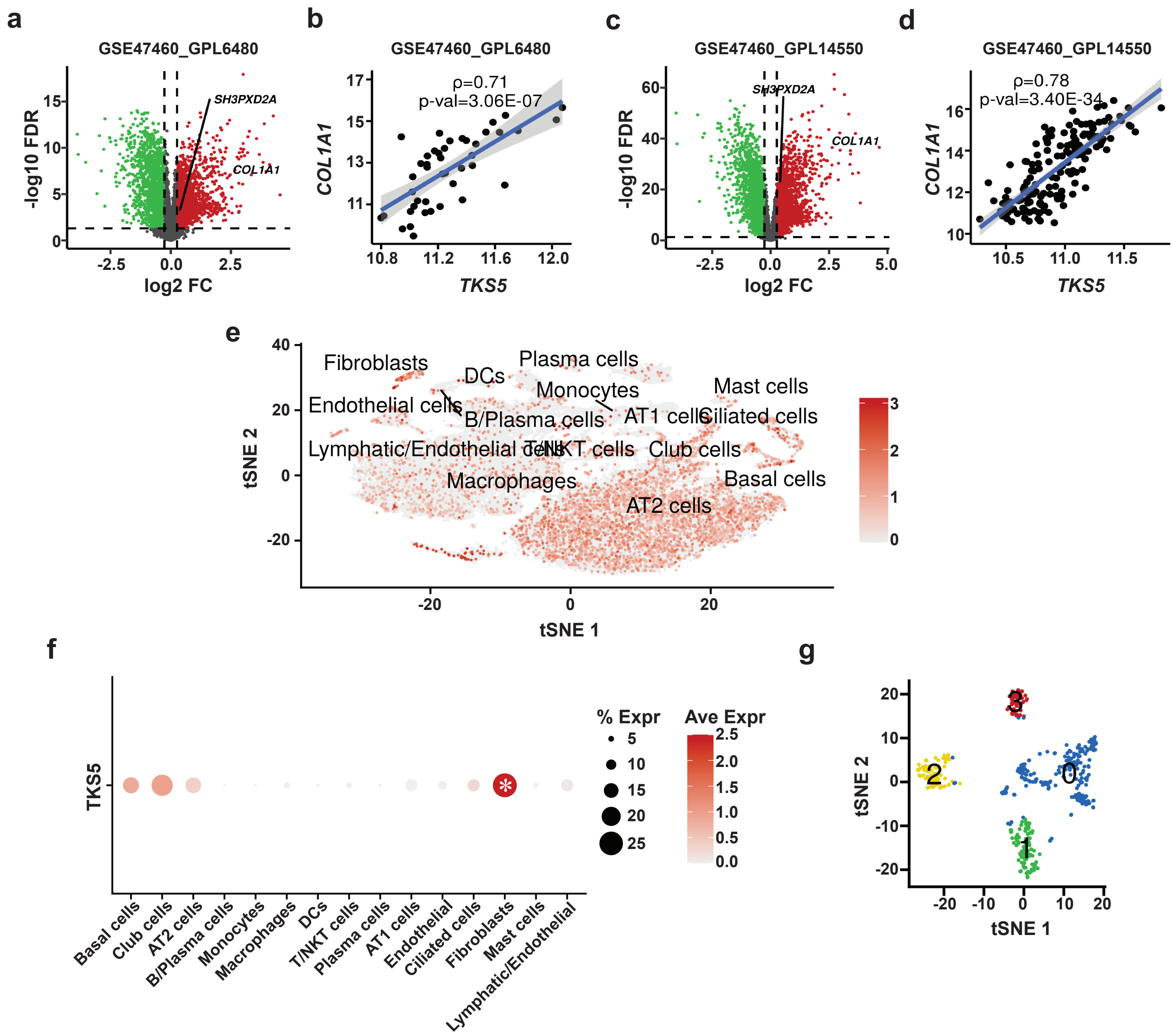

Supplementary Fig. 1. Increased *TKS5* mRNA expression in the lungs of IPF patients. a, c. Differential expression profiling of IPF lung tissue vs controls: two of the largest publicly available datasets (Supplementary Table 1) are shown (FC>1.2, FDR<0.05). **b, d.** Spearman correlation plot of *TKS5* with *COL1A1* expression in the indicated datasets (**a-c**). **e.** Visual representation of *TKS5* abundance in the detected lung tissue cell clusters from reanalysis of the scRNA-seq dataset of (Reyfman, Walter et al. 2019). **f.** In the same dataset, *TKS5* is expressed primarily by fibroblasts as compared to other cells; statistical significance was assessed with the Wilcoxon rank sum test (FC>1.2, Bonferroni adj-p<0.05). **g.** Fibroblast sub-clusters defined from the same data using a resolution of 0.1 on isolated re-processed fibroblasts.

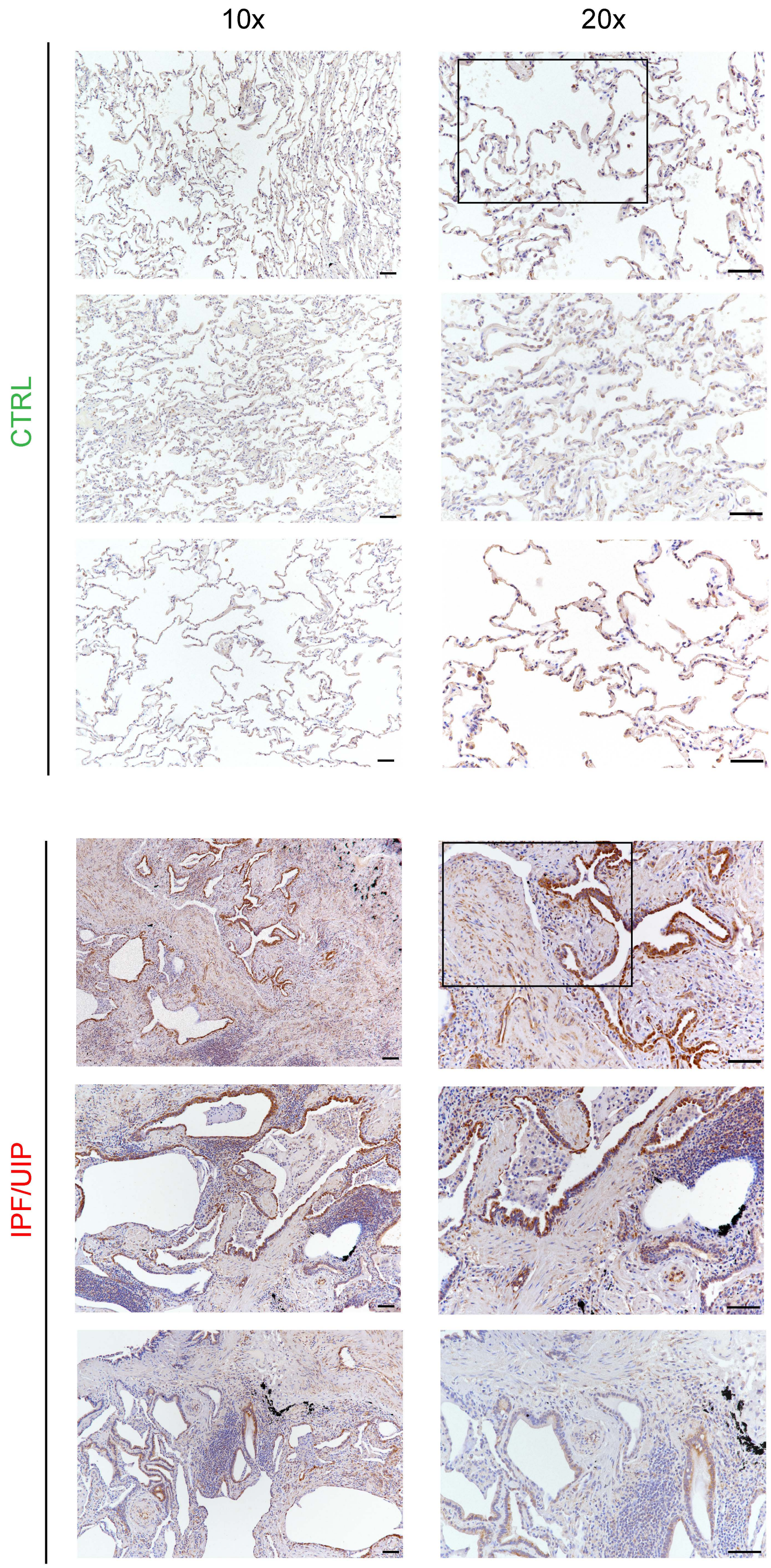

Supplementary Fig. 2. Increased TKS5 immunostaining in the lungs of IPF patients. Images from immunohistochemistry for TKS5 (brown) in fibrotic (IPF/UIP; Idiopathic Pulmonary Fibrosis/Usual Interstisial Pneumonitis) and healthy (CTRL; control) lung tissue; n=3; scale bars=100 µm. The indicated regions are shown in Figure 1e.

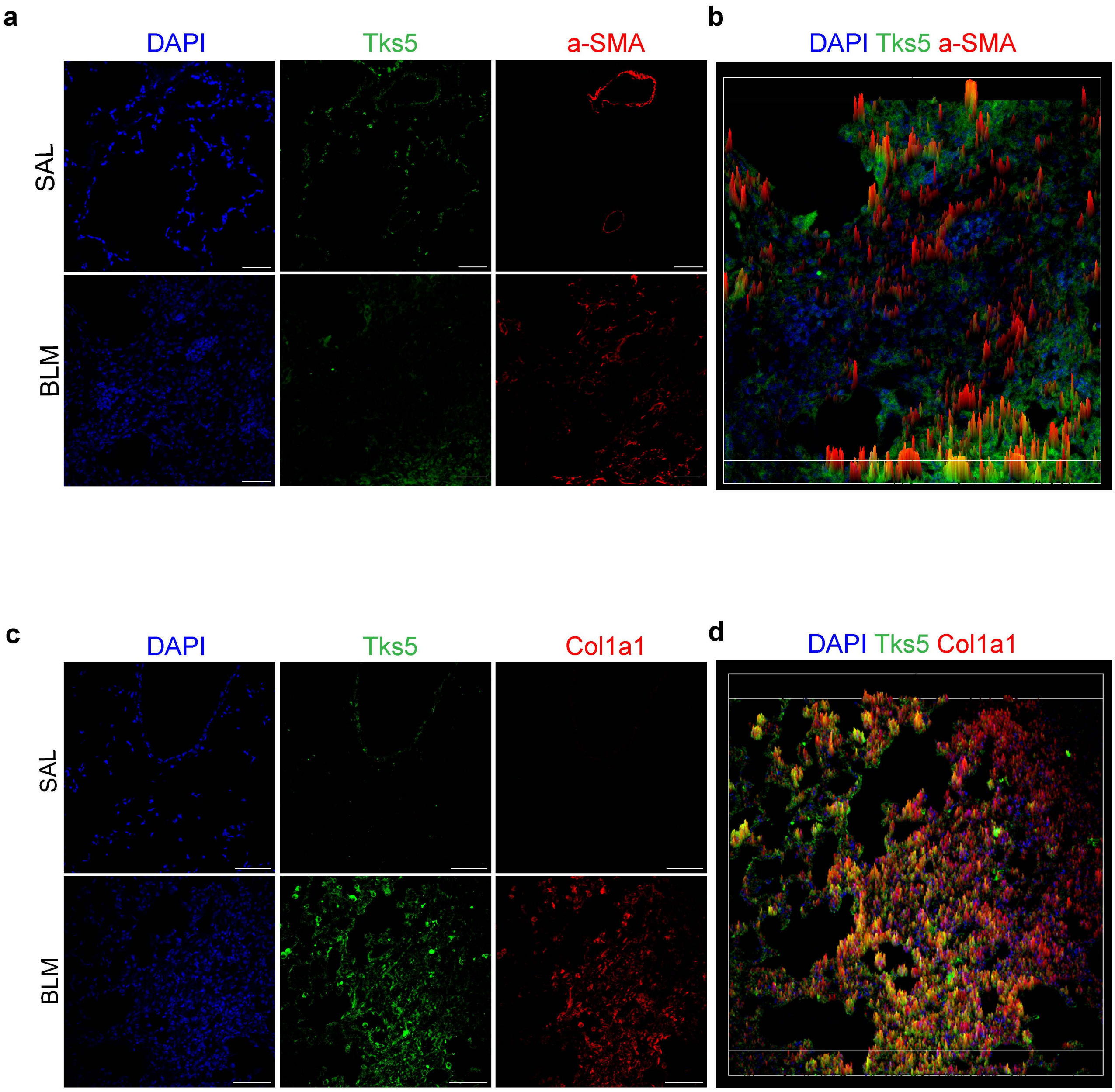

Supplementary Fig. 3. Increased Tks5 expression in the lungs post bleomycin (BLM)-induced pulmonary fibrosis. The individual panels of the corresponding composite images from Fig. 1i against **a.** Tks5/aSMA (Acta2) or **c.** Tks5/Col1a1 (green/red); scale bars=50 µm. **b,d**. 3D surface plots from the merged images of BLM samples as shown in Fig. 1i.

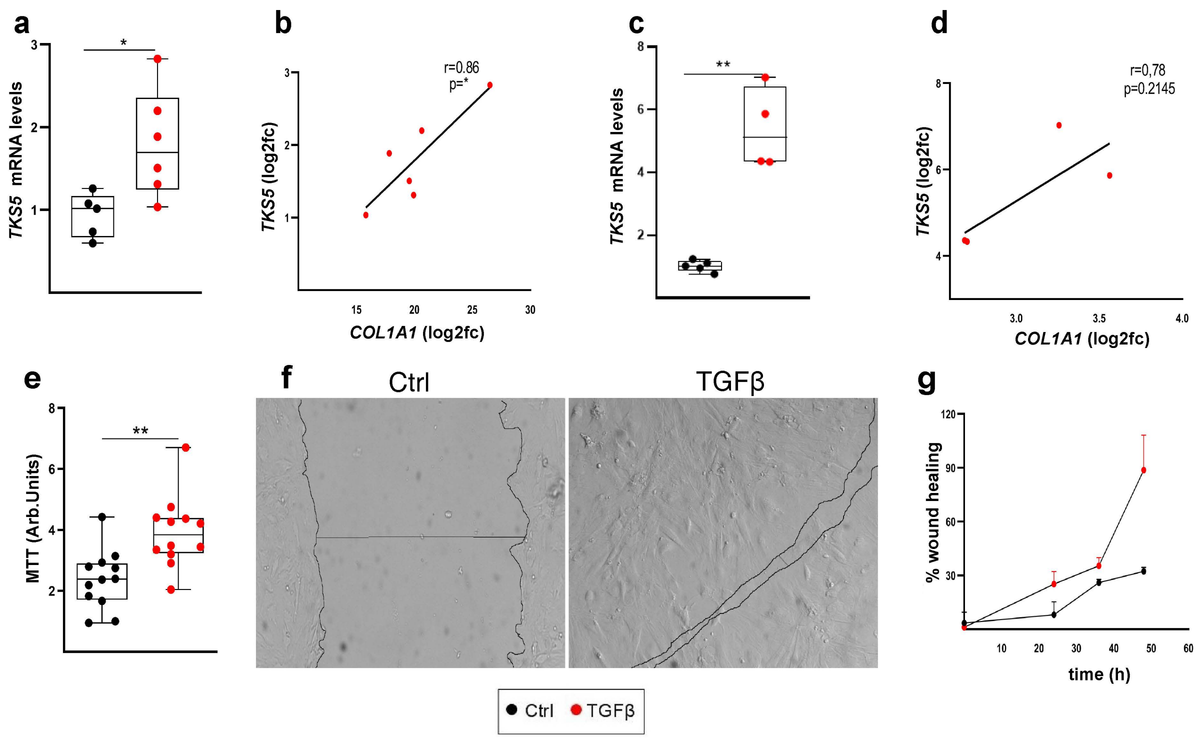

Supplementary Fig. 4. TGFβ stimulates *TKS5* mRNA expression in lung fibroblasts (LFs), correlating with *COL1A1* expression. a. Serum starved primary normal human lung fibroblasts (NHLFs; human lung fibroblasts) were stimulated with recombinant human transforming growth factor β (TGFβ-10 ng/ml for 24 h). TKS5 and COL1A1 mRNA expression was interrogated with Q-RT-PCR (r^2=0.93; E=102%). Values were normalized over the expression of the housekeeping gene *B2M* and presented as fold change over control; n=5/6. Statistical significance was assessed with two tailed t-test; *p=0.023. **b.** Two-tailed pearson correlation plot of *COL1A1* expression in the same samples (*p=0.0286; r=0.86). **c.** Serum starved MRC5 cells (human lung fibroblasts) were stimulated with TGFβ as in a. *TKS5* and *COL1A1* mRNA expression was interrogated with Q-RT-PCR (r^2=0.95; E=97%). Values were normalized over the expression of the housekeeping gene B2M and presented as fold change over control; n=5/4. Statistical significance was assessed with two tailed Welch's test; **p=0.0062. **d.** Two-tailed pearson correlation plot of *COL1A1* expression in the same samples. **e**. TGFβ-induced NHLF proliferation was assessed with the MTT assay; n=12. Statistical significance was assessed with two tailed t-test; **p=0.0017. **f-g**. TGFβ-induced wound healing of NHLFs, as evaluated with the scratch assay. Representative images 48h upon TGFβ stimulation are shown. **g.** Quantification of "wound closure" over time as quantified with Image J. In all panels, representative experiment out of 2 successful independent ones are shown. In all panels all samples are biologically independent; boxplots visualize the median of each distribution; upper/lower hinges represent 1st/3rd quartiles; whiskers extend no further than 1.5 * IQR from the respective hinge. Source data for all panels are provided as a Source Data file.

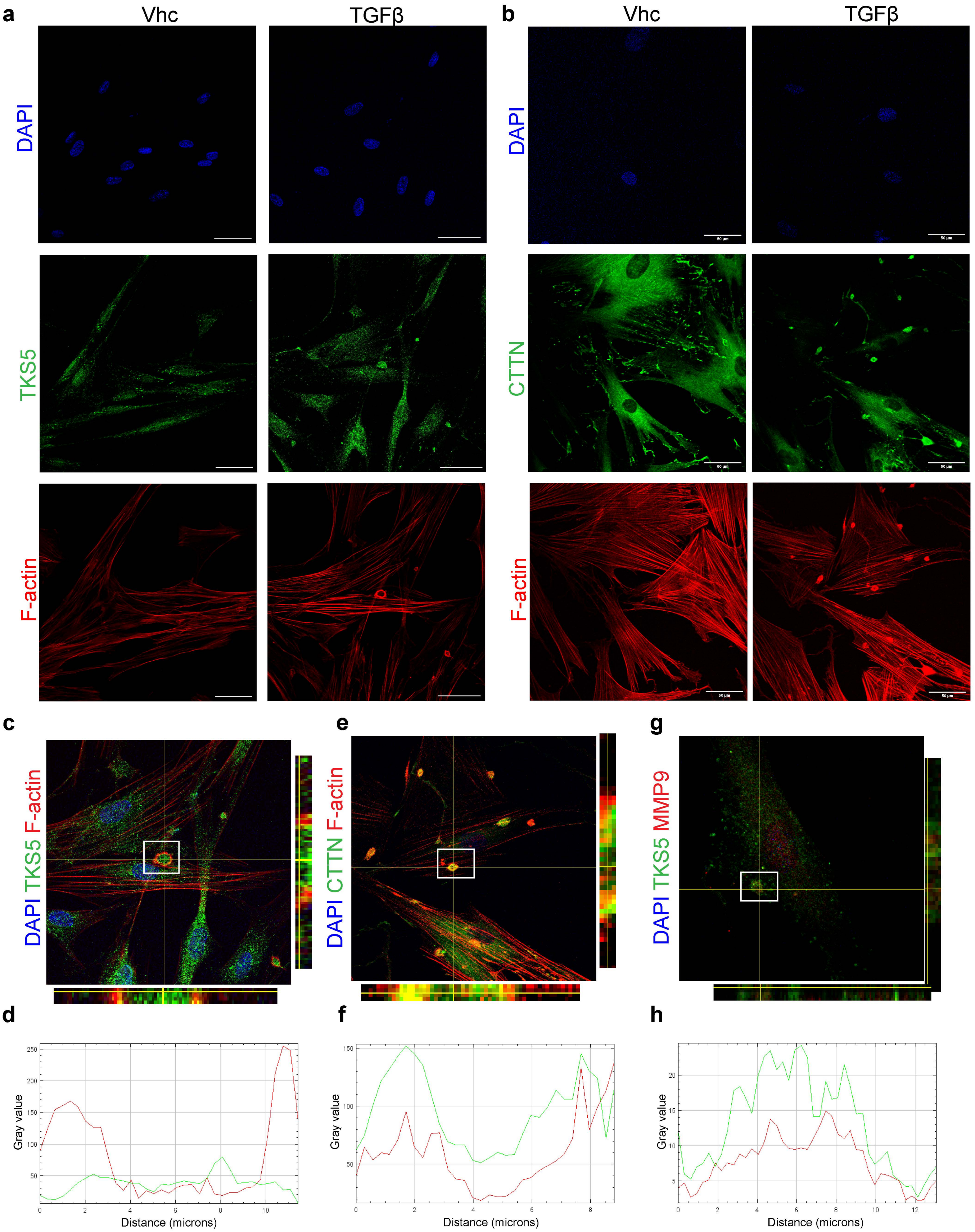

Supplementary Fig. 5. TGF stimulates the formation of podosome rosettes in normal human lung fibroblasts (NHLFs). Serum starved, sub-confluent (70-80%), primary NHLFs were stimulated with recombinant human transforming growth factor β (TGFβ-10 ng/ml for 24h) and immunostained for F-actin and TKS5 **(a)** or CORTACTIN (CTTN) **(b)** (red/green) and counterstained with DAPI. The individual images of the corresponding composite images from Fig.2 c,e are shown; scale bars=50 μm. **c,e,g.** Orthogonal projections of indicated podosomes through z-stacking. **d,f,h** K-curves analysis of the c,e,h images, showing the spatial intensity of fluorescent signals. In all panels, representative experiment out of 3 successful independent ones are shown. In all panels all samples are biologically independent.

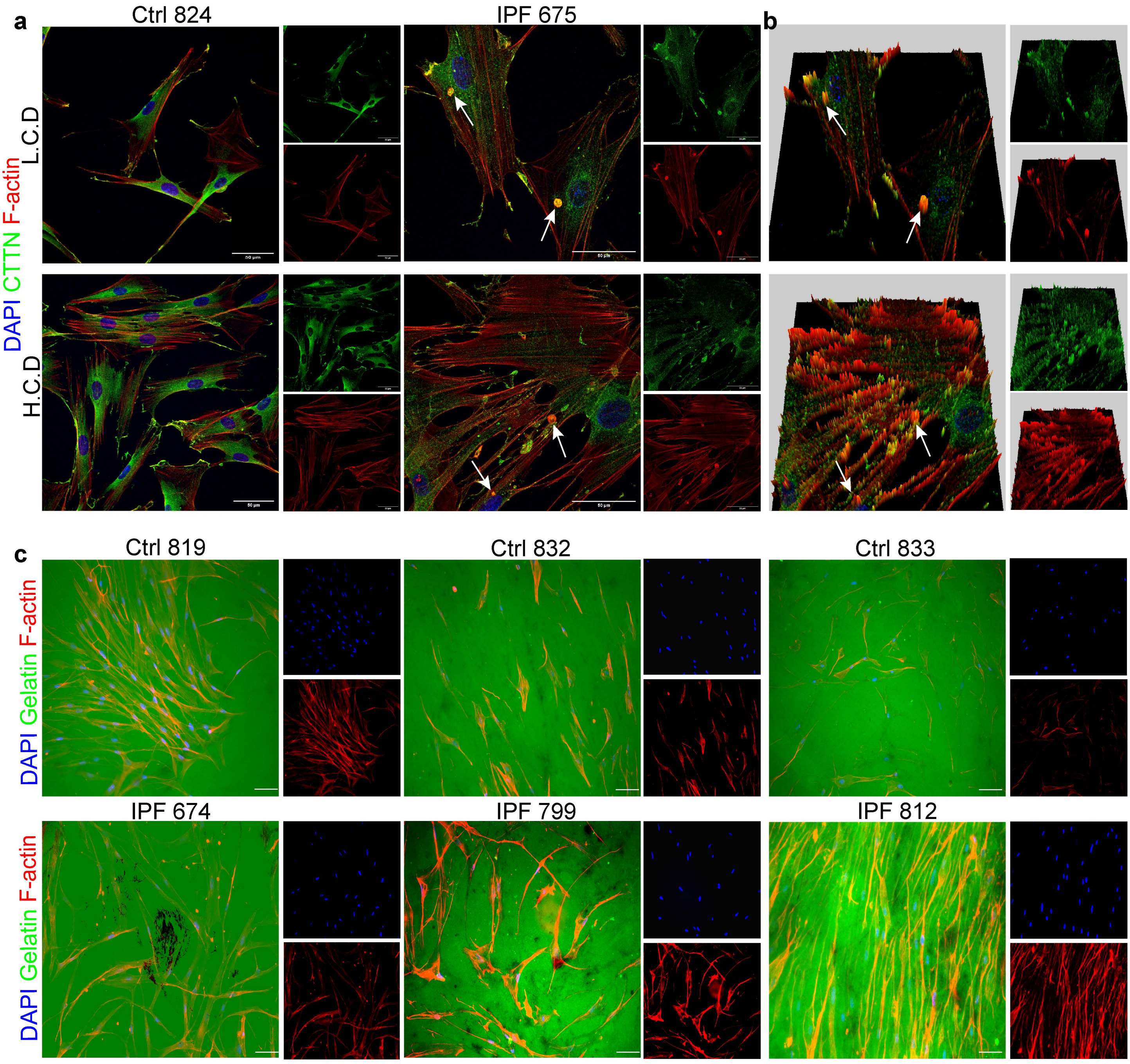

Supplementary Fig. 6. The formation of Extracellular Matrix (ECM) degrading podosome rosettes is an inherent property of IPF human lung fibroblasts (IPF-HLFs). a. Serum starved primary IPF-HLFs and normal human lung fibroblasts (NHLFs) were cultured at low (LCD) or high cell density (HCD) and were immunostained for F-actin and CORTACTIN (CTTN; red/green) and counter stained with DAPI. Representative images are shown; scale bars=50 μm. **b.** 3D surface plots of IPF-HLFs as captured with Image J. **c.** The indicated clones were cultured on a fluorescein-conjugated gelatin substrate (green) and were stained for F-actin (red) and counter stained with DAPI; representative images are shown; scale bars=50 μm.

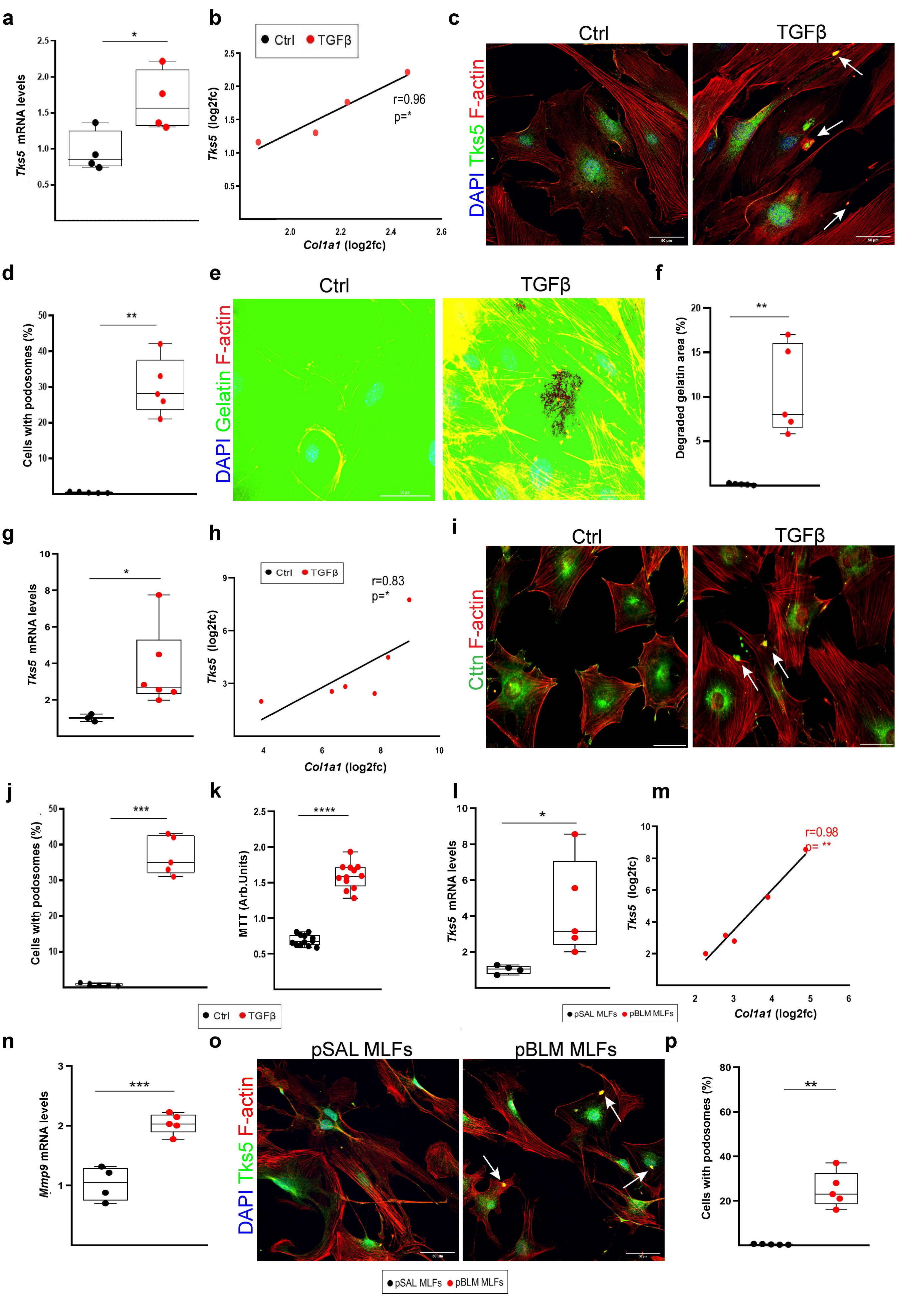

Supplementary Fig. 7. TGFβ-induced, podosome rosettes is an inherent property of post bleomycin (BLM) lung fibroblasts (LFs).

Supplementary Fig. 7. TGFβ-induced, podosome rosettes is an inherent property of post bleomycin (BLM) lung fibroblasts (LFs). Serum starved primary normal mouse lung fibroblasts (NMLFs) or 3T3 cells were stimulated with recombinant transforming growth factor β (TGFβ-10 ng/ml for 24 h). **a-b.** *Tks5* and *Col1a1* mRNA expression was interrogated with Q-RT-PCR in NMLFs. Values were normalized over the expression of the housekeeping gene *B2m* and presented as fold change over control; n=4. Statistical significance was assessed with two tailed t-test; *p=0.0322. **b**. Two-tailed pearson correlation plot of *Col1a1* expression in the same samples (*p=0.0186, r=0.96). **c**. Representative composite images from double immunostaining for F-actin and Tks5 (red/green), counter stained with DAPI; scale bars 50μm; arrows indicate representative podosomes. **d**. Quantification of the number of podosome-containing cells per optical field; n=5. Statistical significance was assessed with two tailed Welch's test; **p=0.0012. **e.** Representative composite images of the TGFβ-induced degradation (black holes) of a fluorescein-conjugated gelatin (green) substrate by NMLFs; scale bars 50μm. **f.** Quantification of gelatin degradation, as quantified with ImageJ; n=5. Statistical significance was assessed with two tailed Welch's test; **p=0.0098. **g-h.** *Tks5* and *Col1a1* mRNA expression were interrogated with Q-RT-PCR in 3T3 cells as described in a-b; n=3/6. Statistical significance was assessed with two tailed Mann-Whitney test; *p=0.0238. **h**. Two-tailed spearman correlation plot of *Col1a1* expression in the same samples (*p=0.0167, r=0.83). **i**. Representative composite images from double immunostaining for F-actin and Cortactin (Cttn; red/green); scale bars 50μm; arrows indicate representative podosomes **j**. Quantification of the number of podosome-containing cells per optical field; n=5. Statistical significance was assessed with two tailed Welch's test; ***p=0.0001. **k.** TGFβ-induced 3T3 cell proliferation was assessed with the MTT assay; n=12. Statistical significance was assessed with two tailed Welch's test (****p<0.0001). **l-m.** *Tks5* and *Col1a1* mRNA expression in mouse LFs isolated post BLM administration were detected with Q-RT-PCR, performed as in a,g; n=4/5. Statistical significance was assessed with two tailed Welch's test; *p=0.0464 **m**. Two-tailed pearson correlation plot of *Col1a1* expression in the same samples (**p=0.0023, r=0.98). **n**. *Mmp9* mRNA levels, quantified as in l; n=4/5. Statistical significance was assessed with two tailed t-test; ***p=0.0003. **o.** Representative composite images from double immunostaining for F-actin and Tks5 (red/green), counter stained with DAPI; scale bars 50μm; arrows indicate representative podosomes. **p.** Quantification of the number of podosome-containing cells per optical field; n=5. Statistical significance was assessed with two tailed Welch's test; **p=0.0023. In all panels, representative experiment out of 2 successful independent ones are shown. In all panels all samples are biologically independent; boxplots visualize the median of each distribution; upper/lower hinges represent 1st/3rd quartiles; whiskers extend no further than 1.5 * IQR from the respective hinge. Source data for all panels are provided as a Source Data file.

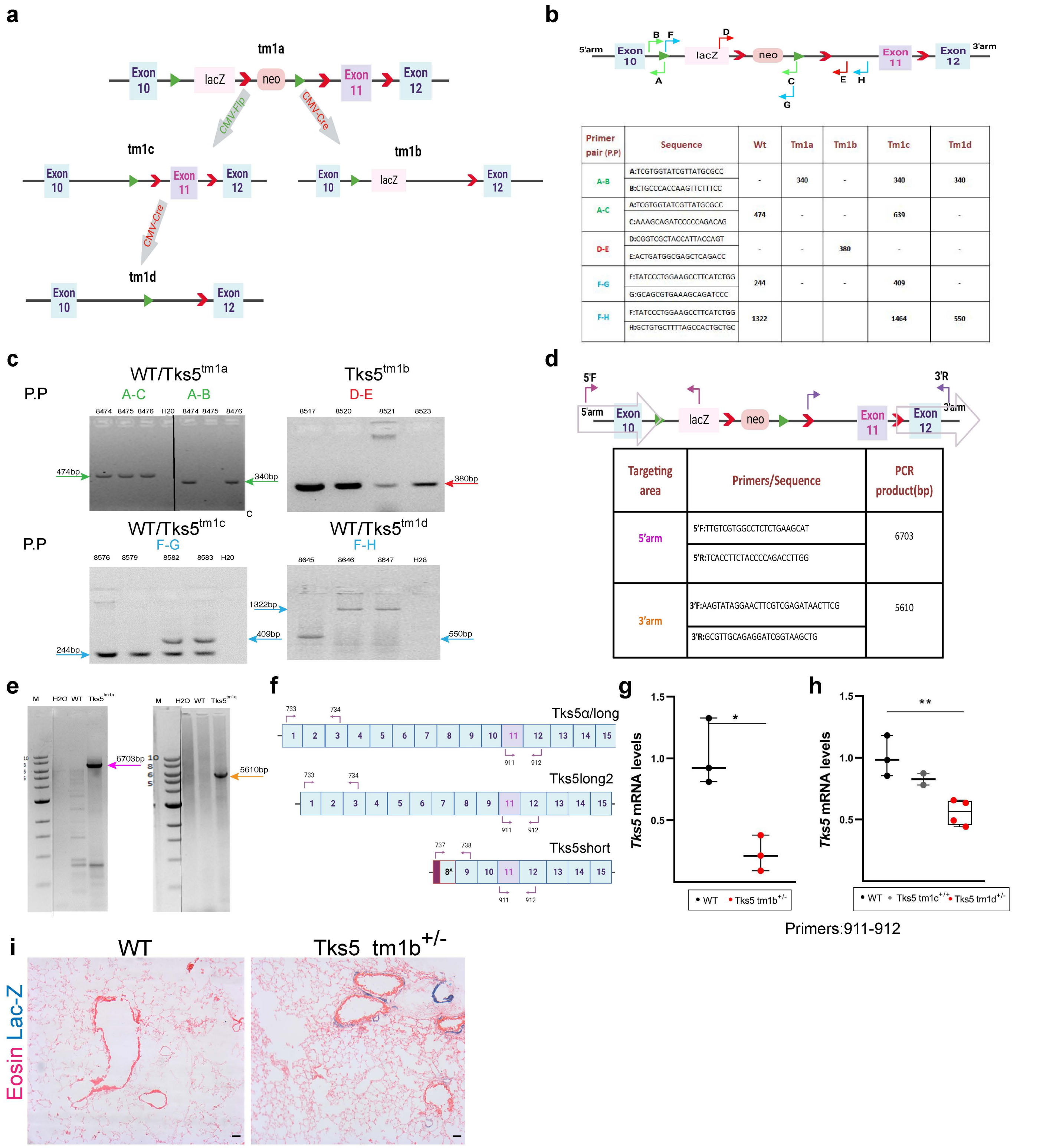

Supplementary Fig. 8. Generation of mouse conditional or obligatory knock out alleles for *Tks5*. a. Schematic presentation of knock out alleles and genetic strategy. **b.** Genotyping strategy, as well as primer sequences and the expected product length of genotyping are indicated. **c.** Confirmation of recombination for all different alleles with genomic PCR. The primer pairs (P.P) used from the Table (b) are also indicated **d.** Genotyping strategy, as well as primer sequences and the expected product length of long-range PCR are indicated. **e.** Confirmation of successful targeting with long range genomic PCR. **f.** Schematic presentation of Tks5 mouse isoforms and the location of the real-time PCR primers, which are used to identify the deletion and the different isoforms. **g.** *Tks5* mRNA levels in lungs of tm1b mice were detected with Q-RT-PCR. Values were normalized over the expression of the housekeeping gene *B2m* and presented as fold change over control; n=3. PCR performed with primers 911-912, detecting the deletion of critical exon 11 from all isoforms. Statistical significance was assessed with two tailed t-test; *p=0.011. **h.** *Tks5* mRNA levels in lungs of tm1d mice were detected as in g; n=3/2/4. Statistical significance was assessed with or two tailed one-way ANOVA; **p=0.0075. **i.** Lac-Z staining (blue) in the lungs from Tks5^{tm1b} mice and control littermates, indicating Tks5 lifelong transcriptional activation; scale bars 50μm. All samples are biologically independent; boxplots visualize the median of each distribution; upper/lower hinges represent 1st/3rd quartiles; whiskers extend no further than 1.5 * IQR from the respective hinge. Source data for panels g-h are provided as a Source Data file.

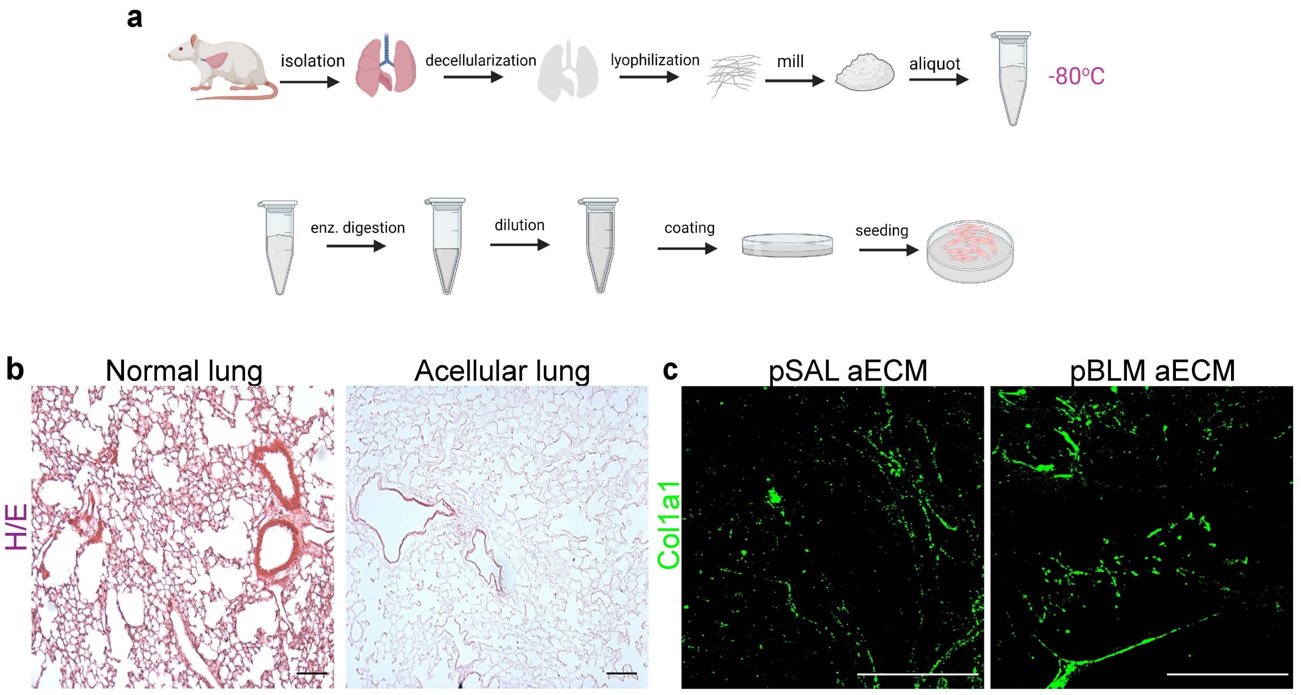

Supplementary Fig. 9. Acellular extracellular matrix (aECM) as a cell substrate for aututologus in vitro culture of lung fibroblasts. a. Schematic presentation of generation of aECM from mouse lungs. **b.** Confirmation of decellularization (absence of cells) with Hematoxylin&Eosin (H&E) staining; scale bars 50μm. **c.** Representative images of immunofluorescent staining for Col1a1 (green) of aECM, generated from lungs upon BLM administration. As expected, aECM generated from mouse lungs post Bleomycin (pBLM), is enriched in Col1a1 compared with the post Saline (pSAL) aECM; scale bars 50μm. Representative experiment out of 2 successful independent ones is shown.

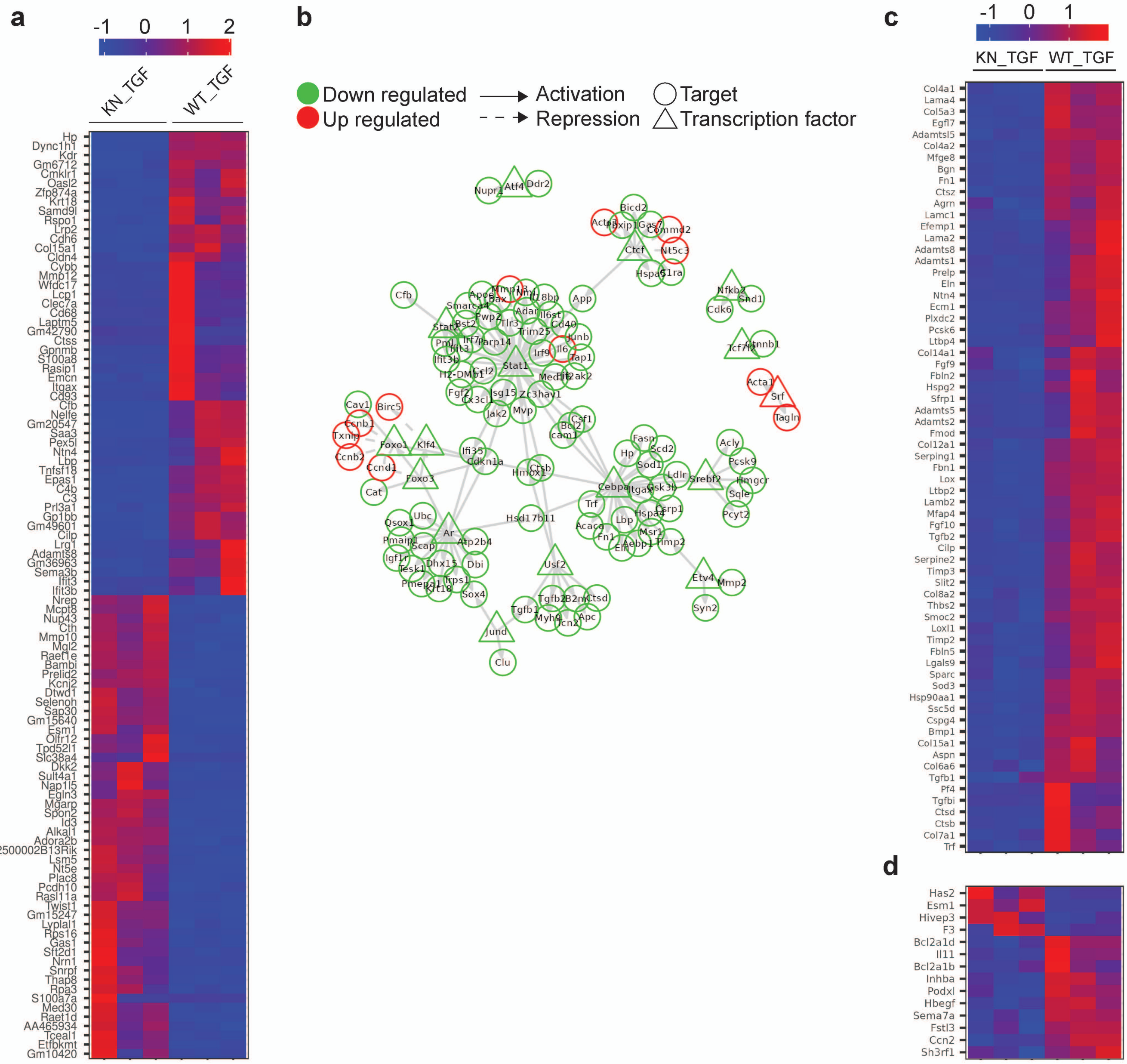

Supplementary Fig. 10. Differential expression analysis of haploinsufficient *Tks5*$^{+/-}$ TGFβ-induced lung fibroblasts (LFs). a. Heatmap of top 100 statistically significant (FC≥ 1.2 Benjamini-Hochberg corrected PANDORA differentially expressed genes (DEGs; $p < 0.05$) in *Tks5*$^{+/-}$ LFs. **b.** Interaction network of differentially expressed transcription factors and their targets in *Tks5*$^{+/-}$ LFs. **c.** Heatmap of the features in the core enrichment set of the GO:0062023 (https://www.ebi.ac.uk/QuickGO/term/GO:0062023) gene ontology term as revealed during pathway analysis. **d.** Heatmap of the deregulated mouse homologous genes of the top 50 up-regulated features in invasive fibroblasts as given in the supplementary data of PMID: 30763282 (https://pubmed.ncbi.nlm.nih.gov/30763282/).

Supplementary Table 1. *TKS5 mRNA* expression in cohorts of IPF patients as compared with the corresponding respective controls. All re-normalized and re-analyzed datasets are sourced from Fibromine (Fanidis, Moulos et al. 2021).

PMID	Samples	GEO dataset	Platform	IPF/Ctrl	FC	FDR
19363140 https://www.ncbi.nlm.nih.gov/pubmed/19363140	Lungs of patients collected during pulmonary transplant compared with control ones derived from cancer free lung margins.	GSE10667 https://www.ncbi.nlm.nih.gov/geo/query/acc.cgi?acc=GSE10667	GPL4133 https://www.ncbi.nlm.nih.gov/geo/query/acc.cgi?acc=GPL4133	21/14	1.29	3.43E-02
30111332 https://www.ncbi.nlm.nih.gov/pubmed/30111332	Fresh frozen lung samples from IPF patients were compared to normal lung tissue flanking pulmonary cancer areas.	GSE110147 https://www.ncbi.nlm.nih.gov/geo/query/acc.cgi?acc=GSE110147	GPL6244 https://www.ncbi.nlm.nih.gov/geo/query/acc.cgi?acc=GPL6244	22/11	-1.23	1.95E-03
21241464 https://www.ncbi.nlm.nih.gov/pubmed/21241464	IPF lung tissue originated from LTRC were assessed in comparison to healthy lung tissue of cancer patients and a lung transplant.	GSE21369 https://www.ncbi.nlm.nih.gov/geo/query/acc.cgi?acc=GSE21369	GPL570 https://www.ncbi.nlm.nih.gov/geo/query/acc.cgi?acc=GPL570	11/6	2.09	4.81E-02
21974901 https://www.ncbi.nlm.nih.gov/pubmed/21974901	IPF lung samples transcriptional profile was compared to that of control samples from lung volume reduction during transplantation.	GSE24206 https://www.ncbi.nlm.nih.gov/geo/query/acc.cgi?acc=GSE24206	GPL570 https://www.ncbi.nlm.nih.gov/geo/query/acc.cgi?acc=GPL570	8/6	2.10	1.08E-02
26560100 https://www.ncbi.nlm.nih.gov/pubmed/26560100	IPF lung samples were compared to healthy ones collected during an exploratory surgery.	GSE47460 https://www.ncbi.nlm.nih.gov/geo/query/acc.cgi?acc=GSE47460	GPL6480 https://www.ncbi.nlm.nih.gov/geo/query/acc.cgi?acc=GPL6480	28/15	1.23	3.69E-03
26560100 https://www.ncbi.nlm.nih.gov/pubmed/26560100		GSE47460 https://www.ncbi.nlm.nih.gov/geo/query/acc.cgi?acc=GSE47460	GPL14550 https://www.ncbi.nlm.nih.gov/geo/query/acc.cgi?acc=GPL14550	84/75	1.31	1.54E-20
21360508 https://www.ncbi.nlm.nih.gov/pubmed/21360508	Microarray data from IPF patients were juxtaposed to data from control individuals.	GSE48149 https://www.ncbi.nlm.nih.gov/geo/query/acc.cgi?acc=GSE48149	GPL16221 https://www.ncbi.nlm.nih.gov/geo/query/acc.cgi?acc=GPL16221	12/5	1.56	2.79E-03
25217476 https://www.ncbi.nlm.nih.gov/pubmed/25217476	Differences between IPF and control transcriptomic profile were examined based on lung biopsies or explants.	GSE53845 https://www.ncbi.nlm.nih.gov/geo/query/acc.cgi?acc=GSE53845	GPL6480 https://www.ncbi.nlm.nih.gov/geo/query/acc.cgi?acc=GPL6480	39/7	1.28	1.24E-02
28230051 https://www.ncbi.nlm.nih.gov/pubmed/28230051	LTRC IPF and control samples were examined to identify transcriptional differences between disease and steady state conditions.	GSE92592 https://www.ncbi.nlm.nih.gov/geo/query/acc.cgi?acc=GSE92592	GPL11154 https://www.ncbi.nlm.nih.gov/geo/query/acc.cgi?acc=GPL11154	19/18	1.78	4.24E-06
29329637 https://www.ncbi.nlm.nih.gov/pubmed/29329637	Scarred regions from explanted lungs were compared to healthy control lung tissues.	GSE99621 https://www.ncbi.nlm.nih.gov/geo/query/acc.cgi?acc=GSE99621	GPL16791 https://www.ncbi.nlm.nih.gov/geo/query/acc.cgi?acc=GPL16791	8/8	1.52	3.70E-03

PMID: PubMed ID; GEO: gene expression database - datasets are hyperlinked; Platform: the hyperlinked numbers indicate the profiling technology; FC: fold change; FDR: false discovery rate; IPF: idiopathic pulmonary fibrosis; LTRC: Lung Tissue Research Consortium.

Supplementary Table 2. Demographics and clinical characteristics of lung tissue donors.

Characteristic	IPF (*n*=20)	Control (*n*=9)	COPD (n=19)
Age (yr)(Mean ± SD)	64.8 ± 8.5	68.8 ± 14.8	67.9 ± 14.5
Sex, *n* (%)			
Males	12 (60%)	5 (55.6%)	15 (79%)
Females	8 (40%)	4 (44.4%)	4 (21%)
Race, *n (%)*			
White	19 (95%)	9 (100%)	19 (100%)
Hispanic	1 (5%)	0	0
Pulmonary function tests (Mean ± SD)			
FVC%	65.8 ± 14.1	93.9 ± 16.8	76.5 ± 14.7
DLCO%	45.6 ± 14.9	85.6± 13.8	58.9 ± 21.7
FEV1%	73.3 ± 14	92.6 ± 12.2	53.3 ± 21.7

**FVC%: Forced vital capacity percent predicted; DLCO%: Carbon monoxide diffusing capacity percent predicted; FEV1% Forced expiratory volume in 1 second percent predicted.*

Supplementary Table 3. Demographics and clinical characteristics of lung fibroblasts donors

Characteristic	IPF (n=5)	Control (n=5)
Age (yr)(Mean ± SD)	66.7± 2.9	64.8 ± 10.3
Sex, *n* (%)		
Males	3 (60%)	3 (60%)
Females	2 (40%)	2 (40%)
Pulmonary function tests (Mean ± SD)		
DLCO%	47.5 ± 12.4	52 ± 12.4
FEV1/FVC%	96 ± 7**	72 ± 10
Podosomes		
% Cells with podosomes/area	51.6 ± 5.7****	13.4 ± 2.5
Podosomes/cell	2.9±0.4****	0.8±0.3

FVC%: Forced vital capacity percent predicted, DLCO%: Carbon monoxide diffusing capacity percent predicted. FEV1% Forced expiratory volume in 1 second percent predicted. Statistical significance was assessed between IPF and Control groups with two-sided unpaired t-test (denote p=0.0029 ****denote p<0.0001).*

Supplementary Table 4. Compounds with a transcriptional profile similar to TGFβ-induced *Tks5*$^{+/-}$ lung fibroblasts.

NCS	Name	Main Target	Targets	Cell line	PMID	IPF Relevance
1.760	GSK-1070916	Aurora kinase	AURKB|AURKC|AURKA|CYP2D6|CYP3A4	BT20	32761869 https://pubmed.ncbi.nlm.nih.gov/32761869/	Inhibition of Aurora Kinase B attenuates fibroblast activation and pulmonary fibrosis
1.733	PSB-069	NTPDase	ENTPD1|ENTPD2|ENTPD3	MCF7	-	-
1.716	SANT-2	Smoothened receptor	SMO|DHH|IHH|PTCH1|SHH	HA1E	-	-
1.697	entinostat	HDAC	HDAC1|HDAC2|HDAC3|HDAC9	YAPC	28315487 https://pubmed.ncbi.nlm.nih.gov/28315487/	Reversion of TGFb1-induced, XPLN/ SPARK-mediated extracellular matrix turnover in human foetal lung fibroblast cell line
1.695	pazopanib	VEGFR |KIT |PDGFR	KDR|KIT|FLT1|FLT4|PDGFRB|PDGFRA|BRAF|CYP2B6|CYP2C8|CYP2E1|DDR2|CSF1R|FGF1|FGFR1|FGFR3|ITK|SH2B3	MCF10A	21992121 https://pubmed.ncbi.nlm.nih.gov/21992121/<br>27279371 https://pubmed.ncbi.nlm.nih.gov/27279371/	Tyrosine kinase inhibitor attenuated worsening of lung function, maintained quality of life and reduced acute exacerbations of IPF patients<br>PDGFR inhibition attenuated enhanced differentiation and proliferation of myofibroblasts
1.683	crizotinib	ALK	ALK|MET|CYP2B6|CYP3A5|MST1R|ROS1	TMD8	15563636 https://pubmed.ncbi.nlm.nih.gov/15563636/	PDGFb1-induced lung fibrosis is blocked by an orally active ALK5 kinase inhibitor
1.671	emetine	Protein synthesis	RPS2	A375	18503048 https://pubmed.ncbi.nlm.nih.gov/18503048/	Increase of Sulf1 mRNA levels, a potential negative regulator of TGFb-1 induced fibrogenesis, through inhibition of protein translation
1.667	TAK-875	Insulin	FFAR1|INS	A375	-	-
1.664	filgotinib	JAK	JAK1|JAK2|JAK3|TYK2	XC.L10	29440315 https://pubmed.ncbi.nlm.nih.gov/29440315/	Block of endothelial to mesenchymal transition and artery smooth muscle cell to myofibroblast transition via JAK inhibition thus reducing bleomycin-induced pulmonary fibrosis
1.664	arofylline	Phosphodiesterase	PDE4A|PDE4B|PDE4C|PDE4D	PC3	-	-
1.663	raloxifene	Estrogen receptor	ESR1|ESR2|ACVRL1|ENG	HT29	35841004 https://pubmed.ncbi.nlm.nih.gov/35841004/	Reduction of systemic sclerosis iPSC-derived fibroblast proliferation, production of extracellular matrix and skin fibrosis in human organoids and mouse model.
1.661	WH-4023	Src	LCK|SRC|ABL1	MCF10A	35998281 https://pubmed.ncbi.nlm.nih.gov/38998281/	Saracatinib, a Selective Src Kinase Inhibitor, Blocks Fibrotic Responses in Preclinical Models of Pulmonary Fibrosis
1.661	brefeldin-a	Protein synthesis |BIG1	ARFGEF1|ARFGEF2|GBF1|ARF1|CYTH2	HCT116	8429043 https://pubmed.ncbi.nlm.nih.gov/8429043/	Inhibition of intracellular degradation and thus secretion of collagen in normal human fetal lung fibroblasts
1.655	TGX-221	PI3K	PIK3CB|PIK3CD	MCF10A	21984893 https://pubmed.ncbi.nlm.nih.gov/2184893/	PI3K inhibition attenuates myofibroblast differentiation in the human lungs
1.654	motesanib	KIT |PDGFR |VEGFR	FLT1|FLT4|KDR|KIT|PDGFRA|RET	MCF10A	-	-

NCS: Normalized connectivity score; PMID: PubMed ID; IPF: Idiopathic pulmonary fibrosis

Supplementary Table 5. Primers and sequences used for RT- and genomic PCR.

	Description	Sequence- Lot (for taqman)
**Human**	m1 SH3PXD2A	Hs00206037
	m1 B2M	Hs00984230
	B2M F	AGATGAGTATGCCTGCCGTG
	B2M R	CTGCTTACATGTCTGGATCCCA
	TKS5 LONG F	CTCCCAAGAAGGACGTGACA
	TKS5 LONG R	CTCTTGGACACTTCCCCAGT
	COL1A1 F	CGAAGACATCCCACCAATCAC
	COL1A1 R	CATCGCACAACACCTTGCC
**Mouse**	B2m F	TTCTGGTGCTTGTCTCACTGA
	B2m R	CAGTATGTTCGGCTTCCCATTC
	Tks5 F	GGAGCCCCTCTAAACACTATGT
	Tks5 R	GGCCACCTTCAATAGGAAACTT
	Tks5 long F	TTATCAACGTGACCTGGTCTG
	Tks5 long R	TTCGGATCCTTCTGGCCAC
	Tks5short F	TGGCTCACCGCGTGCTTTCTG
	Tks5 short R	CCTTGCTCTTCAGATGTGCTCACAA
	Tks5 tm1b-d (ex11-12) F	AAGACGAGATCGGCTTCGAG
	Tks5 tm1b-d (ex11-12) R	TCCCTATGATCTCCACCGGA
	Col1a1 F	CTACTACCGGGCCGATGATG
	Col1a1 R	CGATCCAGTACTCTCCGCTC
	Mmp9 F	CTGGACAGCCAGACACTAAAG
	Mmp9 R	CTCGCGGCAAGTCTTCAGAG
**Mouse genomic primers**	(A) CAS_R1_Term	TCGTGGTATCGTTATGCGCC
	(B) Sh3pxd2a 270538 F	CTGCCCACCAAGTTCTTTCC
	(C) Sh3pxd2a 270538 R	AAAGCAGATCCCCCAGACAG
	(D) Tm1b prom F	CGGTCGCTACCATTACCAGT
	(E) Floxed LR	ACTGATGGCGAGCTCAGACC
	(F) Sh3pxd2a F	TATCCCTGGAAGCCTTCATCTGG
	(G) Sh3pxd2a R1	GCAGCGTGAAAGCAGATCCC
	(H) Sh3pxd2a R2	GCTGTGCTTTTAGCCACTGCTGC
	Long range 5' arm F	TTGTCGTGGCCTCTCTGAAGCAT
	Long range 5' arm R	TCACCTTCTACCCCAGACCTTGG
	Long range 3' arm F	AAGTATAGAACTTCGTCGAGATAACTTCG
	Long range 3' arm R	GCGTTGCAGAGGATCGGTAAGCTG
