# Supplementary material for: SRC and TKS5 mediated podosome formation in fibroblasts promotes extracellular matrix invasion and pulmonary fibrosis
Source: Nat Commun. 2023 Sep 21;14:5882. doi: 10.1038/s41467-023-41614-x (PMC10514346; doi:10.1038/s41467-023-41614-x)
Supplement: Supplementary file 3 — Description of Additional Supplementary Files Document [file 41467_2023_41614_MOESM3_ESM.pdf]

Description of Additional Supplementary Files.

Supplementary Data 1: Deregulated genes upon Tks5 haploinsufficiency and treatment with TGFb

Supplementary Data 2: Deregulated pathways upon Tks5 haploinsufficiency and treatment with TGFb

Supplementary Movie 1 - The formation of podosomes is an inherent property of Idiopathic Pulmonary Fibrosis (IPF) lung fibroblasts. Primary human lung fibroblasts isolated from an IPF patient, were cultured in vitro and were stained for F-actin (red),cortactin (green) and DAPI (blue).
