# Supplementary material for: SRC and TKS5 mediated podosome formation in fibroblasts promotes extracellular matrix invasion and pulmonary fibrosis
Source: Nat Commun. 2023 Sep 21;14:5882. doi: 10.1038/s41467-023-41614-x (PMC10514346; doi:10.1038/s41467-023-41614-x)
Supplement: Supplementary file 6 — Reporting Summary [file 41467_2023_41614_MOESM6_ESM.pdf]

nature portfolio

Corresponding author(s): V.Aidinis

Last updated by author(s): Sep 7, 2023

Reporting Summary

Nature Portfolio wishes to improve the reproducibility of the work that we publish. This form provides structure for consistency and transparency in reporting. For further information on Nature Portfolio policies, see our Editorial Policies and the Editorial Policy Checklist.

Statistics

For all statistical analyses, confirm that the following items are present in the figure legend, table legend, main text, or Methods section.

n/a	Confirmed	
☐	☒	The exact sample size (*n*) for each experimental group/condition, given as a discrete number and unit of measurement
☐	☒	A statement on whether measurements were taken from distinct samples or whether the same sample was measured repeatedly
☐	☒	The statistical test(s) used AND whether they are one- or two-sided<br>*Only common tests should be described solely by name; describe more complex techniques in the Methods section.*
☒	☐	A description of all covariates tested
☐	☒	A description of any assumptions or corrections, such as tests of normality and adjustment for multiple comparisons
☐	☒	A full description of the statistical parameters including central tendency (e.g. means) or other basic estimates (e.g. regression coefficient) AND variation (e.g. standard deviation) or associated estimates of uncertainty (e.g. confidence intervals)
☐	☒	For null hypothesis testing, the test statistic (e.g. *F*, *t*, *r*) with confidence intervals, effect sizes, degrees of freedom and *P* value noted<br>*Give P values as exact values whenever suitable.*
☒	☐	For Bayesian analysis, information on the choice of priors and Markov chain Monte Carlo settings
☒	☐	For hierarchical and complex designs, identification of the appropriate level for tests and full reporting of outcomes
☐	☒	Estimates of effect sizes (e.g. Cohen's *d*, Pearson's *r*), indicating how they were calculated

Our web collection on statistics for biologists contains articles on many of the points above.

Software and code

Policy information about availability of computer code

Data collection	Histology images were captured by a Q Imaging EXI Aqua digital camera using the Q-Capture Pro 7 software.
Data analysis	Single cell RNA-seq data were processed using Seurat R package (v.3.1.2 & 4.0.5). Initial analysis of sequencing data was performed in the Ion Torrent server. Quant-Seq (Lexogen) sequencing data initial analysis was performed in the Ion Torrent server. Then FASTQ files were pre-processed with Trim Galore (v.0.6.51) and subsequently mapped to GRCm38 reference genome using HISAT2 (v.2.2.1) and BOWTIE2 (v.2.3.5.1) aligners. Downstream analysis of BAM files was performed using metaseqR2 R package (v.1.9.2). Other R packages used for performed in-silico analyses are clusterProfiler (v.4.0.5), XML (v.3.99.0.8), rentrez (v.1.2.3), pubmed.mineR (v.1.0.19), biomaRt (v. 2.48.3) and DoRothEA (v.1.4.2). L1000 data from Expanded CMap LINCS Resource 2020 (last update 11/23/2021) were queried and processed by cmapR R package (v.1.4.0) for purposes of Connectivity Map analysis. Prism (GraphPad 8.0.1) software was used for statistical analyses.

For manuscripts utilizing custom algorithms or software that are central to the research but not yet described in published literature, software must be made available to editors and reviewers. We strongly encourage code deposition in a community repository (e.g. GitHub). See the Nature Portfolio guidelines for submitting code & software for further information.

Data

Policy information about availability of data

All manuscripts must include a data availability statement. This statement should provide the following information, where applicable:

- Accession codes, unique identifiers, or web links for publicly available datasets
- A description of any restrictions on data availability
- For clinical datasets or third party data, please ensure that the statement adheres to our policy

All re-analyzed publicly available datasets are listed in Supplementary Table 1, including accession numbers and hyperlinks. Quant-Seq data have been deposited at GEO database under the accession code GSE220982. Already published, re-analyzed single cell RNA-seq data used are available at GEO database under the accession code GSE122960. All other relevant experimental data are within the paper and its supplementary information files. Data and code for the recreation of the computationally-created figures of the paper have been deposited at Zenodo with DOI: 10.5281/zenodo.8296510 and at https://github.com/dfanidis/TKS5_podosomes_IPF.

Research involving human participants, their data, or biological material

Policy information about studies with human participants or human data. See also policy information about sex, gender (identity/presentation), and sexual orientation and race, ethnicity and racism.

Reporting on sex and gender	Both males and females were randomly assigned to sex-matched experimental groups. No sex/gender bias has been reported for the used animal models, hence no separate sex/gender analysis has been performed, per common practices in the field.
Reporting on race, ethnicity, or other socially relevant groupings	N/A
Population characteristics	The covariate - relevant population characteristics (age, disease or sex) of human patients (lung tissue and fibroblast donors) are included in Supplementary Tables 2 and 3 respectively.
Recruitment	Patients were recruited according to predefined inclusion and exclusion criteria in the study protocols listed below, that cannot/do not have any impact on presented results or their interpretation. All used human samples are subsets of previously collected and/or reported samples, selected for this study solely on availability and confirmed disease status, thus excluding any self-selection or other biases.
Ethics oversight	All studies with human patient samples were performed in accordance with the Helsinki Declaration principles. Lung tissue samples (Supplementary Table 2) were obtained through the University of Pittsburgh Health Sciences Tissue Bank and Yale University Pathology Tissue service, a subset of previously well characterized and published samples62; studies had been approved by the Yale University Institutional Review Board (Yale IRB). Lung fibroblasts were isolated from the lung tissue of IPF patients and from the adjacent healthy tissue of patients undergoing open lung surgery for cancer (Supplementary Table 3) at the Department of Pulmonology, Bichat-Claude Bernard Hospital, Paris/France; studies were approved by the Committee for Personal Protection (CPP) - Ile de France 1 (#0911932). All patients consented in writing to the use of their samples for research purposes; no compensation was provided for their participation.

Note that full information on the approval of the study protocol must also be provided in the manuscript.

Field-specific reporting

Please select the one below that is the best fit for your research. If you are not sure, read the appropriate sections before making your selection.

☒ Life sciences ☐ Behavioural & social sciences ☐ Ecological, evolutionary & environmental sciences

For a reference copy of the document with all sections, see nature.com/documents/nr-reporting-summary-flat.pdf

Life sciences study design

All studies must disclose on these points even when the disclosure is negative.

Sample size	Sample size was determined based on previous studies and by using power analysis (R package pwr).
Data exclusions	No data were excluded.
Replication	All presented experimental data have been duplicated. Shown results are representative of at least 2 successful repetitions that all yielded similar results.
Randomization	Human patients were classified to groups based on their disease. The medians for sex and age of each group have been reported.<br><br>wt and genetically modified littermate mice were randomly assigned to sex-matched experimental groups.

Blinding	The investigators were blinded to group allocation during the initial data collection and analysis.

Reporting for specific materials, systems and methods

We require information from authors about some types of materials, experimental systems and methods used in many studies. Here, indicate whether each material, system or method listed is relevant to your study. If you are not sure if a list item applies to your research, read the appropriate section before selecting a response.

Materials & experimental systems

n/a	Involved in the study
☐	☒ Antibodies
☐	☒ Eukaryotic cell lines
☒	☐ Palaeontology and archaeology
☐	☒ Animals and other organisms
☒	☐ Clinical data
☒	☐ Dual use research of concern
☒	☐ Plants

Methods

n/a	Involved in the study
☒	☐ ChIP-seq
☒	☐ Flow cytometry
☒	☐ MRI-based neuroimaging

Antibodies

Antibodies used	Primary antibodies:anti-Tks5 (SH3 domain) rabbit monoclonal antibody (Merck, 3174822, 1:100), anti-SH3PXD2A mouse monoclonal antibody (Origene, clone OTI1F5-TA811757S, LOT F001, 1:250), col1a1 rabbit polyclonal antibody (Invitrogen, PA5-29569, LOT XK3738717 1:100), anti-A-actin (sma) mouse monoclonal antibody, (Origene, clone UM870129, LOT F001 1:250), recombinant Anti-Cortactin rabbit monoclonal (EP1922Y) antibody (Abcam, ab81208, 1:500), Alexa Fluor™ 633 Phalloidin (Invitrogen, A22284, LOT 2274768 1:50), MMP-9 XP Rabbit monoclonal (D6O3H) Antibody (Cell signaling #13667, LOT 3 1:100).<br><br>Secondary antibodies: Goat anti-Rabbit IgG (H+L) Cross-Adsorbed Secondary Antibody Alexa Fluor 488 (Life technologies, A11008, LOT1470706), Goat anti-Rabbit IgG (H+L) Cross-Adsorbed Secondary Antibody, Alexa Fluor 555 (Life technologies, A21428, LOT 1670185), Goat anti-Mouse IgG (H+L) Highly Cross-Adsorbed Secondary Antibody Alexa Fluor 488 (Life technologies, A11029, LOT1705900) Goat anti-Mouse IgG (H+L) Highly Cross-Adsorbed Secondary Antibody Alexa Fluor 555 (Life technologies, A22424, LOT1726548); all secondary antibodies were used at a 1:500 dilution.
Validation	All antibodies were selected from previous publications, while all antibodies were validated by the corresponding manufacturers for the specific employed methodologies (IHC/IF).

Eukaryotic cell lines

Policy information about cell lines and Sex and Gender in Research

Cell line source(s)	NIH/3T3 is a fibroblast cell line that was isolated from a mouse NIH/Swiss embryo. Purchased from ATCC (#CRL-1658).<br><br>MRC-5 is a diploid cell line made up of fibroblasts isolated from the lung tissue derived from a White, male, 14-week-old embryo by J.P. Jacobs in 1966. Purchased from ATCC (#CCL-171).
Authentication	Cell lines have been recently purchased from ATCC for this project. Morphology and doubling times of purchased cell lines were identical to the ATCC provided info.
Mycoplasma contamination	Cell lines were purchased mycoplasma free. No signs of mycoplasma contamination, based on the unchanged proliferation/ metabolic profile of the cells as tested frequently with the MTT assay.
Commonly misidentified lines (See ICLAC register)	No commonly misidentified cell lines were used in the study.

Animals and other research organisms

Policy information about studies involving animals; ARRIVE guidelines recommended for reporting animal research, and Sex and Gender in Research

Laboratory animals	Mice were bred at the animal facilities of Biomedical Sciences Research Center 'Alexander Fleming', under SPF conditions, at 20–22° C,55 ±5% humidity, and a 12-h light-dark cycle; food and water were provided ad libitum. All experimentation was performed with 8-10-week-old C57Bl6/J mice.
Wild animals	No wild animals were used in the study.
Reporting on sex	Both males and females were randomly assigned to sex-matched experimental groups. No sex bias has been reported for the used animal models, in the reported experimental conditions, hence no separate sex analysis has been performed.

Field-collected samples	No field collected samples were used in the study.
Ethics oversight	All experimentation was approved by the Institutional Animal Ethical Committee (IAEC) of Biomedical Sciences Research Center "Alexander Fleming", as well as by the Veterinary Service of the governmental prefecture of Attica, Greece (# 8441/2017).

Note that full information on the approval of the study protocol must also be provided in the manuscript.
